# Cumulative exposure to traumatic events and craving among women in residential treatment for substance use disorder: The role of emotion dysregulation and mindfulness disposition

**DOI:** 10.3389/fpsyg.2022.1048798

**Published:** 2022-11-24

**Authors:** Mariana Sanchez, Hortensia Amaro

**Affiliations:** ^1^Department of Health Promotion & Disease Prevention, Robert Stempel College of Public Health and Social Work, Florida International University, Miami, FL, United States; ^2^Herbert Wertheim College of Medicine, Florida International University, Miami, FL, United States

**Keywords:** trauma, women, emotion dysregulation, craving, SUD, mindfulness

## Abstract

**Objective:**

Strong evidence links exposure to traumatic life events with subsequent substance use disorders (SUD). Compared to men, women in SUD treatment are more likely to have a history of trauma, characterized in part by emotion dysregulation known to negatively influence treatment outcomes. Existing research has been conducted with predominantly male and non-Hispanic White samples, with an emphasis on adverse childhood experiences. Little is known about how exposure to cumulative traumatic events across the lifespan affects emotion dysregulation and how this may influence craving, particularly among racial and ethnic minoritized women with SUD. Mindfulness disposition may serve as a protective factor that could buffer the impact of trauma exposure and emotion dysregulation on substance use craving among underrepresented minoritized women with SUD. This study examined the association between cumulative exposure to traumatic events, emotion dysregulation, and mindfulness disposition on substance use craving in a sample of predominantly Hispanic and non-Hispanic Black women in residential treatment for SUD.

**Method:**

Cross-sectional data were analyzed for a baseline sample of 241 women (56.96% Hispanic, 20.7% non-Hispanic White, 20.7% non-Hispanic Black; age: *M* = 32.11) entering a SUD residential treatment facility who agreed to participate in a parent randomized controlled trial.

**Results:**

Findings indicated that greater cumulative exposure to traumatic events and emotion dysregulation were associated with higher levels of craving. Cumulative traumatic event exposure was indirectly associated with higher craving *via* lower levels in the mindfulness dimension of acting with awareness. Interaction effects also revealed greater exposure to traumatic events was associated with higher levels of craving among women with low and average (but not high) levels of mindfulness disposition.

**Conclusion:**

These findings provide insight into the potential benefits of targeting emotion regulation and mindfulness-building strategies such as acting with awareness in interventions among racial-ethnically diverse women with SUD. These strategies may be particularly beneficial among those that have experienced extensive histories of trauma exposure. Overall, knowledge gained from the present study can be particularly valuable towards informing treatment models in minoritized groups that currently experience disparities in treatment utilization and outcomes.

## Introduction

Substance use disorders (SUD) have been defined as a chronic relapsing condition (McLellan et al., 2005), in which approximately 50% of users relapse within 6 months of treatment completion (McKay and Weiss, 2001). Further, up to 80% of those who relapse experience cycles of treatment, relapse, and problematic use (Scott et al., 2005).

Craving, the subjective urge or desire to consume substances over a long time (Kozlowski and Wilkinson, 1987), strongly predicted relapse across all major drugs of abuse (Serre et al., 2015) in investigations ranging from neurological to psychosocial (Donovan and Witkiewitz, 2012; Witkiewitz et al., 2013b; Moore et al., 2014). As such, craving is a particularly useful target for SUD interventions that aim to reduce the likelihood and severity of relapse (Witkiewitz and Bowen, 2010).

### Craving, trauma, and emotion regulation

Strong evidence links exposure to traumatic life events with subsequent SUD, as indicated by disproportionately higher rates of trauma among substance abusers (Ghorbani et al., 2019; Giarratano et al., 2020; Prangnell et al., 2020). Compared to men, women in SUD treatment are more likely to have a history of trauma, characterized in part by emotion dysregulation known to negatively influence treatment outcomes (Walitzer and Dearing, 2006; Greenfield et al., 2007). Emotion dysregulation has been posited as one mechanism whereby trauma exposure is linked with SUD. As conceptualized by Gratz and Roemer (Gratz and Roemer, 2004), emotion dysregulation is a multifaceted construct consisting of six dimensions: a lack of emotional awareness, limited access to emotion regulation strategies, nonacceptance of emotional responses, difficulties controlling impulses, lack of emotional clarity, and difficulties engaging in goal-directed behaviors. Emotion dysregulation is particularly evident among individuals with SUD and a history of childhood abuse (Ghorbani et al., 2019). Indeed, among individuals in SUD treatment, higher emotion dysregulation has been found among those with a history of childhood trauma exposure, compared to those without such exposure (Weiss et al., 2013).

Notably, most of the research in this area has focused on interpersonal trauma such as exposure to emotional and physical abuse. Much less is known about cumulative lifetime effects of multiple and intersecting traumatic experiences such as exposure to incarceration, discrimination, violent crimes, serious economic instability, and homelessness. Exposure to these traumatic experiences tend to co-occur in the lives of women and can make them particularly vulnerable to SUD (Amaro et al., 2021).

Associations among trauma exposure, emotion dysregulation, and substance use behaviors including craving have also been identified (Szasz et al., 2012; Tull et al., 2016; Garland et al., 2019; Ghorbani et al., 2019; Khosravani et al., 2019). Although growing evidence suggests emotion dysregulation is a possible mediating mechanism between trauma and craving (Ghorbani et al., 2019), the bulk of this research has been conducted with predominantly male, non-Hispanic White and college samples, with an emphasis on adverse childhood experiences (Szasz et al., 2012; Tull et al., 2016; Garland et al., 2019; Ghorbani et al., 2019; Khosravani et al., 2019). Little is known about how exposure to traumatic events across the lifespan affects emotion dysregulation and how this may influence craving, particularly among racial and ethnic minoritized populations with SUD. This knowledge can be particularly valuable towards informing treatment models in minoritized groups that often experience substantial disparities in treatment utilization and outcomes (Guerrero et al., 2013; Pinedo, 2019). Additionally, there is a limited understanding in the current literature base regarding the distinct dimensions of emotion dysregulation that may be linked to craving among women with extensive histories of trauma exposure. This knowledge can be vital in designing SUD treatments for women that are tailored to promote skills in emotion regulation.

### Mindfulness disposition, emotion regulation, and craving

Mindfulness has been defined as the awareness that results from paying attention on purpose, in the present moment, and nonjudgmentally (Kabat-Zinn, 2009). There is growing evidence for the salutary health effects of mindfulness, including its protective effects on substance use behaviors (Bowen et al., 2014; Karyadi et al., 2014; Tomlinson et al., 2018). A meta-analysis by Karyadi et al. (2014) revealed a significant negative association between mindfulness disposition and substance use (i.e., higher levels of trait mindfulness were associated with lower levels of substance use), with larger effects among problematic (vs. nonproblematic) substance use behaviors and for inpatient (vs. outpatient and nonclinical) samples.

Mindfulness has been conceptualized and investigated as both a state (modifiable characteristic) and a trait (i.e., stable characteristic; Tomlinson et al., 2018). The present study focused on trait mindfulness, also known as mindfulness disposition. Mindfulness disposition refers to an individual’s tendency to maintain awareness in a nonjudgmental and nonreactive way to present situations (Carpenter et al., 2019). Mindfulness disposition is thought to be composed of five facets: ability to observe internal and external experiences (observe), acting with awareness in the present moment (awareness), ability to describe internal experiences (describe), not judging inner experiences (nonjudgment), and letting go of thoughts and feelings rather than reacting (nonreactivity Bohlmeijer et al., 2011). Levels of mindfulness disposition vary across the general population, regardless of mindfulness practice (Kabat-Zinn, 2009). Nevertheless, mindfulness disposition can be enhanced through mindfulness training (Kiken et al., 2015). Current theory suggests that including mindfulness training in SUD treatment can improve outcomes by altering the craving–use relationship (Brewer et al., 2013; Elwafi et al., 2013; Witkiewitz et al., 2013a). Mindfulness practices have the potential to increase the ability of individuals in SUD treatment to respond to the unpleasant physical, affective, and cognitive experiences of craving, in nonreactive ways and resist engaging in substance-seeking behavior to alleviate the discomfort (Witkiewitz et al., 2013a,b).

A critical theoretical component of mindfulness is the ability to better regulate responses to emotional experiences. Scholars have posited that key aspects of mindfulness such as present moment awareness and nonjudgmental acceptance result in greater ability to regulate emotions (Teper et al., 2013). Mindfulness practices have also been found to increase emotion regulation and connectivity in areas of the brain associated with these behaviors in populations with SUD (Tang et al., 2016). Taken together, these findings suggest that mindfulness disposition may serve as a protective factor that buffers the impact of trauma exposure and emotion dysregulation on alcohol and drug craving among women with SUD.

The present study examined the associations among cumulative exposure to traumatic events (CETE), emotion dysregulation, mindfulness disposition, and craving in a racially and ethnically diverse sample of women in residential SUD treatment. There is a paucity of knowledge about how these processes function among women with SUD, especially those from diverse racial and ethnic backgrounds (Greenfield et al., 2007). Indeed, underserved groups such as racial-ethnic minoritized populations have not been well represented in the mindfulness research, and particularly as it relates to SUD (Spears, 2019). What is established is that Hispanics and Blacks with SUD are less likely to successfully complete SUD treatment than non-Hispanic Whites (Jacobson et al., 2007; Guerrero et al., 2013) and have inequitable exposure to the criminal justice and child welfare systems (Amaro et al., 2005, 2021; Jones et al., 2011). Indeed, these findings underscore the long-standing need for greater attention to the integration of racial and ethnic minorities in SUD research, the importance of addressing the cumulative effects of traumatic exposure, and the identification of barriers and effective strategies for reducing disparities during SUD treatment (Institute of Medicine US, 2003; National Institute on Drug Abuse, 2016–2020). In an effort to address some of these gaps, the present study pursues the following aims as stated below.

### Research aims

**Figure 1:**
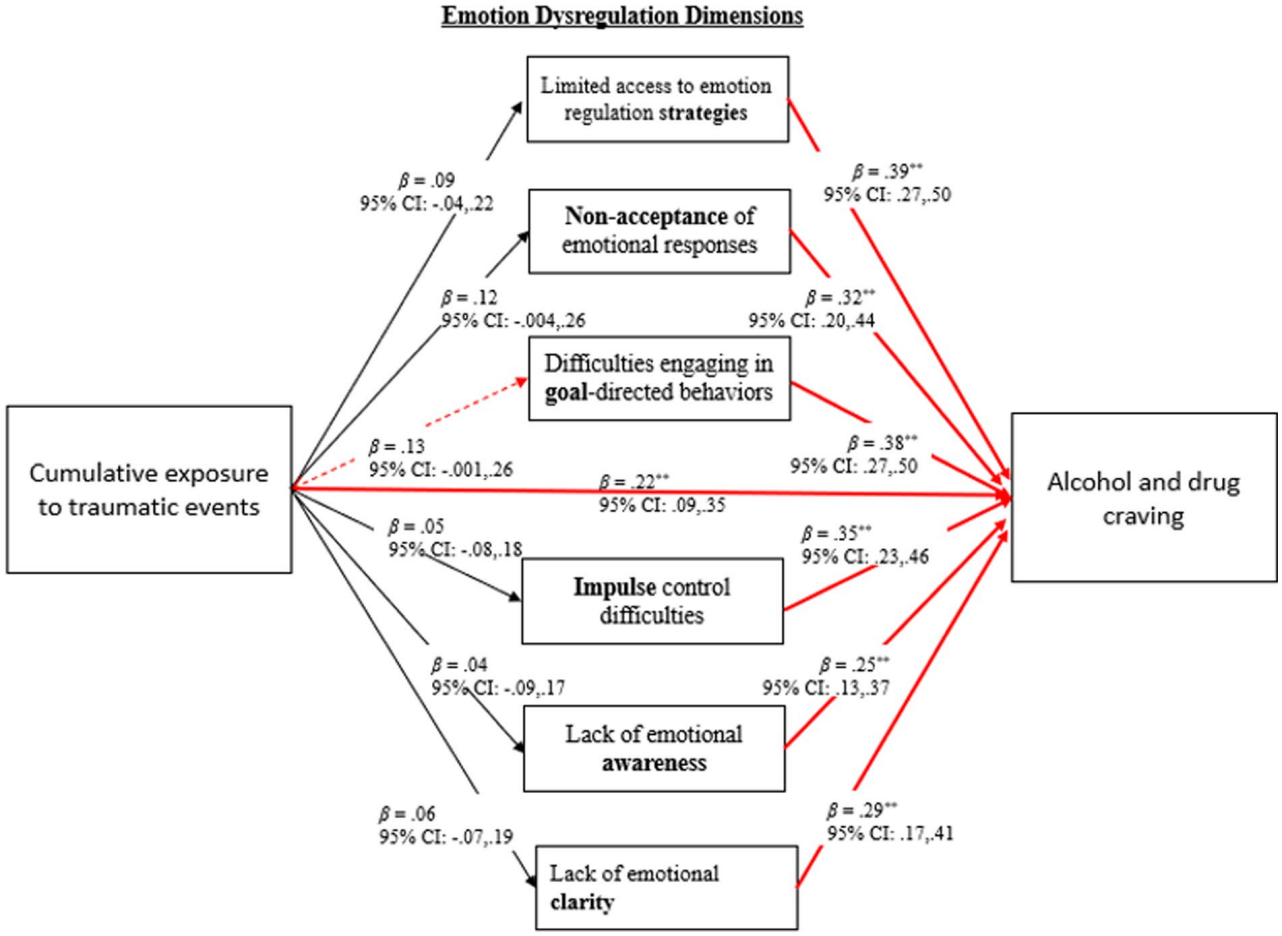
Results for direct and indirect associations between cumulative exposure to traumatic events, emotion dysregulation, and craving. *p < 0.05; **p < 01; Dashed red line denotes marginal significance at *p* = 0.05; Models controlled for race-ethnicity.

Aim 1: Examine the direct and indirect associations between CETE, emotion dysregulation and alcohol and drug craving (see Figure 1).

*Hypothesis 1*: CETE is positively associated with emotion dysregulation and craving.

*Hypothesis 2*: Emotion dysregulation is positively associated with craving.

*Hypothesis 3*: CETE is indirectly associated with craving *via* higher emotion dysregulation.

**Figure 2:**
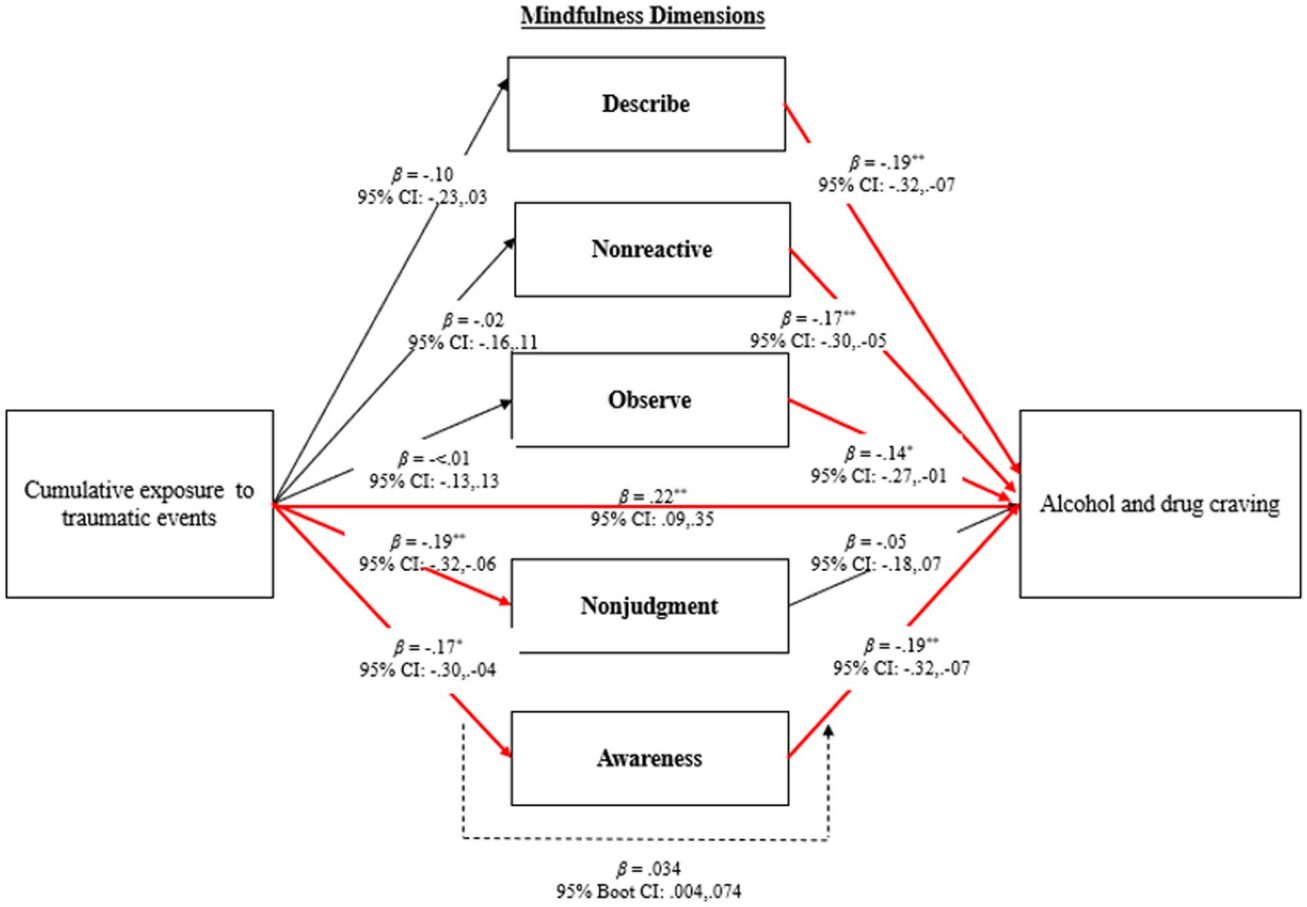
Results for direct and indirect associations between cumulative exposure to traumatic events, mindfulness, and craving. *p < 0.05; **p < 01; Models controlled for race-ethnicity.

Aim 2: Determine the direct and indirect associations between mindfulness disposition, CETE, and craving (see Figure 2).

*Hypothesis 4*: CETE is negatively associated with mindfulness disposition.

*Hypothesis 5*: Mindfulness disposition is negatively associated with craving.

*Hypothesis 6*: CETE is indirectly associated with craving *via* lower levels of mindfulness disposition.

**Figure 3:**
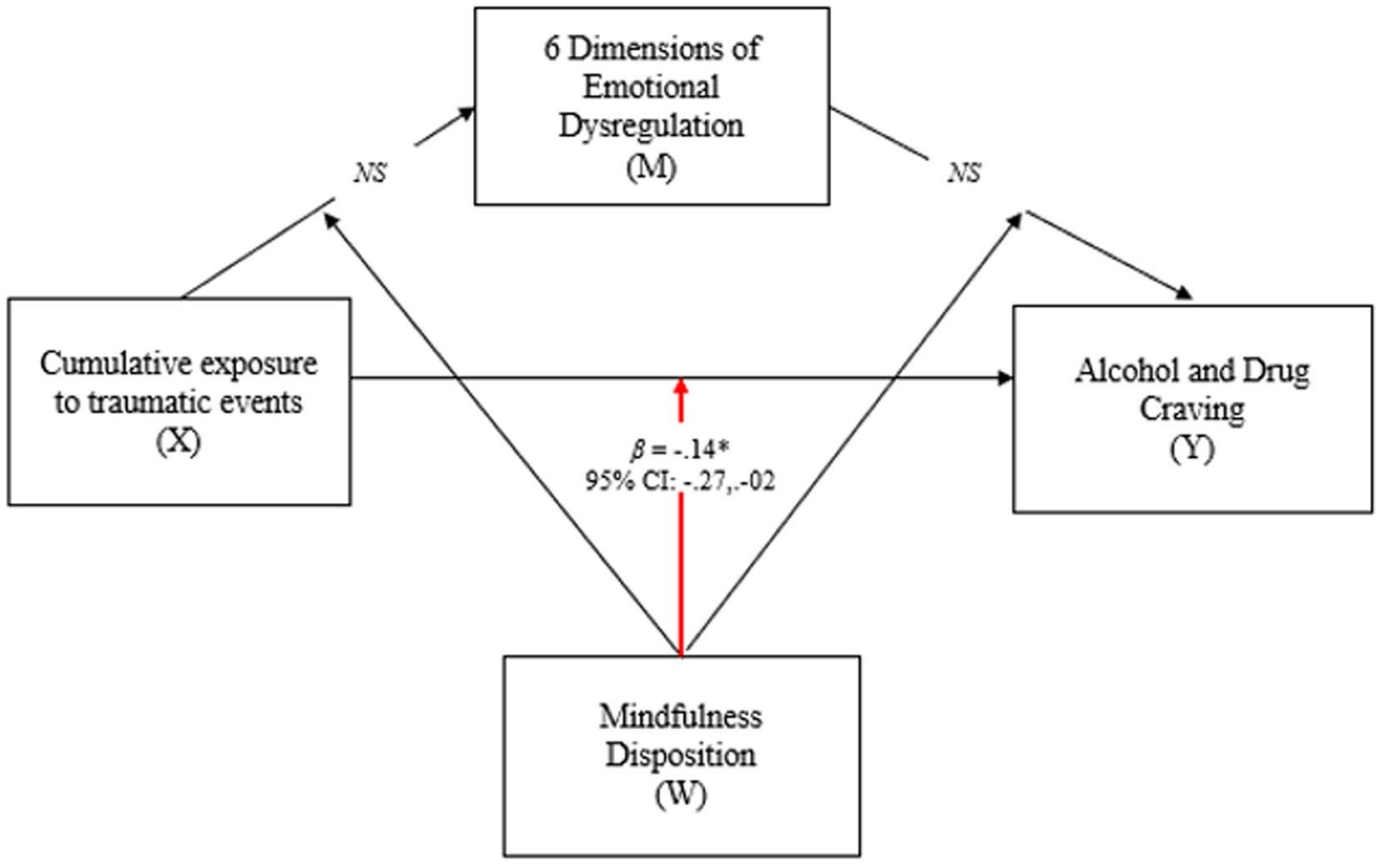
Results for moderating effects of mindfulness disposition on the associations between cumulative exposure to traumatic events, emotion dysregulation, and craving. *p < 0.05; NS = non-significant; X = predictor; M = mediator; Y = outcome; W = Moderator; Model controlled for race-ethnicity.

Aim 3: Test the moderating effect of mindfulness disposition on the associations among CETE, emotion dysregulation, and craving (see Figure 3).

*Hypothesis 7*: Mindfulness disposition moderates the associations between CETE, emotion dysregulation, and craving, such that the relationships between (a) CETE and craving, (b) CETE and emotion dysregulation, and (c) emotion dysregulation and craving are stronger among women reporting low levels of mindfulness disposition.

**Figure 4:**
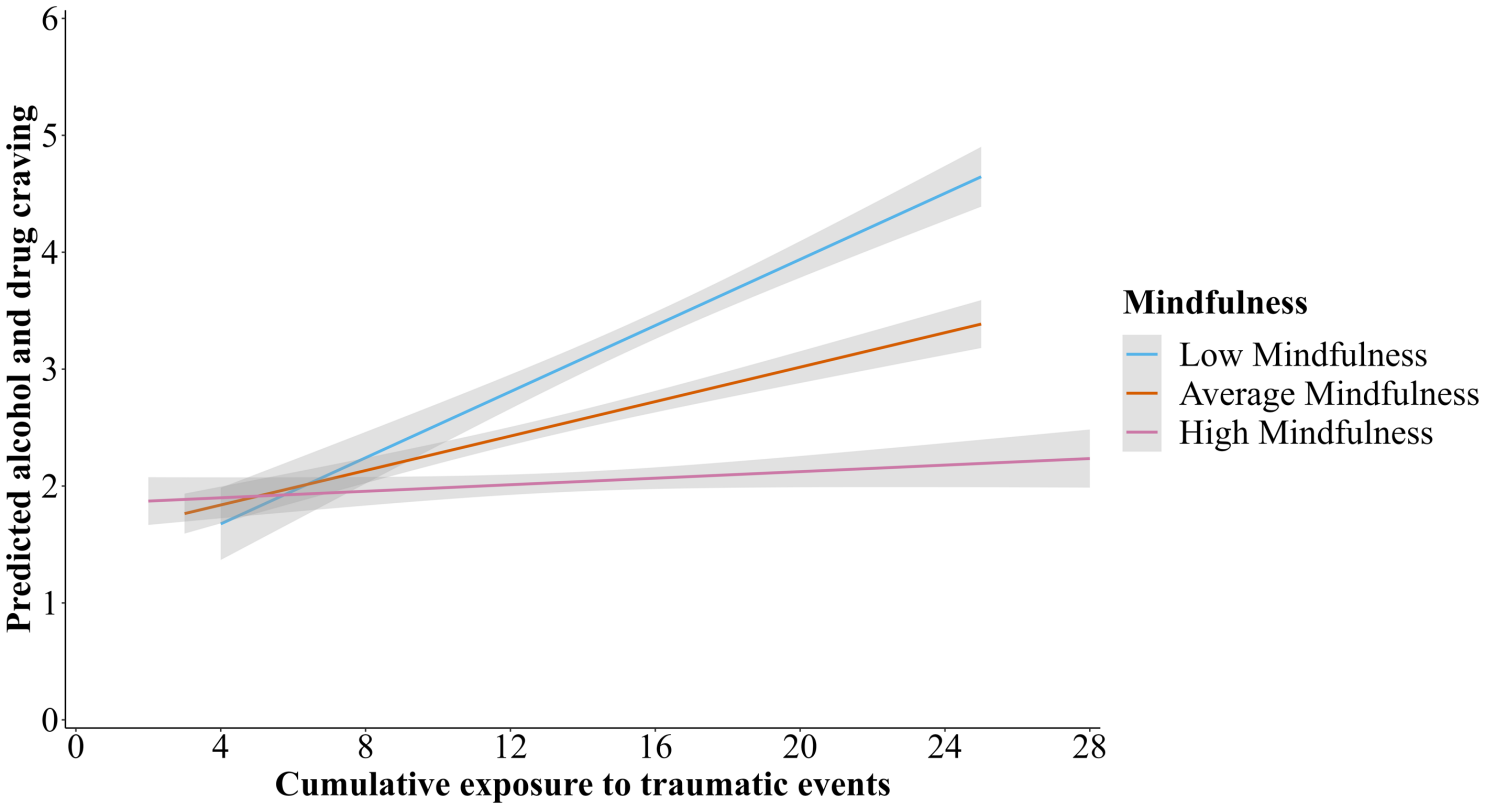
Two-way interaction with mindfulness disposition moderating the association between cumulative exposure to traumatic events and craving.

## Materials and methods

### Participants

Data for the present study came from the baseline interview of a larger randomized clinical trial of Amaro and Black (2021), a mindfulness-based relapse prevention adjunctive intervention program for women (*N* = 245) in residential SUD treatment. Participants in the parent study were recruited at treatment entry and assessed early in the treatment process. Approximately 2–3 weeks after residential treatment entry, the names of clients who assented to be contacted by the research team were provided to the research interviewer. The research interviewer made appointments with prospective participants, conducted the informed consent and HIPAA processes, and were administered the baseline assessment. All data used in the present study was collected during the baseline interview. Inclusion criteria were: aged 18–65, SUD diagnosis, ability to provide consent (i.e., English fluency, no cognitive impairment), and willingness to be audio recorded and provide broad consent for other studies using the collected data. Exclusion criteria included conditions for which the intervention was contraindicated or that could potentially interfere with the intervention (i.e., suicidal ideation in the last 30 days, untreated psychotic disorder, or other untreated chronic serious mental health disorder), current imprisonment, or advanced pregnancy. See Amaro Black (2017) a full description of study methods. Data were collected *via* in-person interviews by a trained research staff using Research Electronic Data Capture. All parent study procedures were approved by the University of Southern California Institutional Review Board (IRB; UP-14-00391).

### Measures

#### Cumulative exposure to traumatic events

The Life Stressor Checklist-Revised (Wolfe and Kimerling, 1997) is a 30-item measure of traumatic events and stressors (with yes-or-no responses). Items include various aspects of traumatic exposure, including but not limited to interpersonal abuse, exposure to violent crimes and the criminal justice system, and homelessness. The scale has been validated among women with comorbid SUD and mental health disorders with histories of interpersonal violence victimization (McHugo et al., 2005). In the present study, we used the sum of dichotomously scored responses (range: 0–30), reflecting events at any point in the lifetime.

#### Emotion dysregulation

The Difficulties in Emotion Regulation Scale (Gratz and Roemer, 2004), a 36-item self-report scale (rated from 1 to 5), examines six dimensions of emotion regulation problems, with higher scores indicating greater emotion dysregulation. In the present study, internal consistency of the full scale (*α* = 0.96) and subscales (nonacceptance, goals, impulse, awareness, strategies, and clarity: 0.89, 0.84, 0.86, 0.82, 0.90, and 0.77, respectively) were adequate.

#### Alcohol and drug craving

The Penn Alcohol Craving Scale (Flannery et al., 1999), a 5-item self-report measure, was adapted to include craving for alcohol and other drugs. The scale measures frequency, intensity, and duration of craving, as well as an overall rating of craving for the previous week, with higher scores reflecting greater craving. Internal consistency in the current sample was excellent (*α* = 0.93).

#### Mindfulness disposition

The Five Facet Mindfulness Questionnaire (Bohlmeijer et al., 2011) short version is a 24-item scale assessing the tendency to be mindful in everyday life. Items are rated on a 5-point Likert scale (1 = *never true* to 5 = *very often true*) and assess five mindfulness dimensions, with higher scores indicating greater mindfulness disposition. All five subscales and the overall scale had adequate internal consistency in this sample (Cronbach’s *α*: observe = 0.72, describe = 0.72, act aware = 0.81, nonjudgment = 0.73, nonreactivity = 0.66, overall = 0.81).

#### Covariates

The following covariates were assessed: age, race and ethnicity (1 = *non-Hispanic White*, 2 = *non-Hispanic Black*, 3 = *Hispanic*), marital status (1 = *married or common law*, 2 = *separated, divorced, or widowed*), education (1 = *less than high school*, 2 = *high school or equivalent*, 3 = *some postsecondary education*), housing in the 8 months prior to treatment (1 = *homeless*, 2 = *non-stable accommodation*, 3 = *institution*, 4 = *own place*, 5 = *someone else’s place*) and employment in the 8 months prior to treatment entry (1 = *full time*, 2 = *part time*, and 3 = *not employed*).

#### Analytic plan

Correlations were computed to assess the unadjusted zero-order relationships between all key observed variables in the research model. Potential covariates were race and ethnicity, age, education, housing, marital status, and alcohol and drug addiction severity in 30 days prior to treatment. One-way analysis of variance was used to evaluate the potential influence of categorical covariates (race and ethnicity, education, housing, employment, and marital status).

Primary data analysis consisted of mediation analyses, wherein bootstrap resampling techniques (*k* = 10,000) were used to generate 95% bias-corrected confidence intervals (CIs) of indirect effects using PROCESS version 3.2 for SPSS 25. We tested CETE’s direct and indirect influence on craving *via* emotion dysregulation. Six individual models were tested (one for each emotion dysregulation dimension). Indirect effects of CETE on craving *via* mindfulness disposition were also tested. Five individual models were conducted (one for each mindfulness disposition dimension). Bias-corrected CIs that did not include zero for the indirect effect indicated statistical significance.

PROCESS version 3.2 was used to conduct moderation models to test whether mindfulness disposition moderated associations between (a) CETE and craving, (b) CETE and emotion dysregulation, and (c) emotion dysregulation and craving (see Figure 2). All analyses controlled for significant covariates (race and ethnicity). No missing data were evident in the primary analysis.

## Results

### Preliminary analyses

The analytic sample (*n* = 241) excluded four participants from the original study sample who identified as “other” race and ethnicity besides the three primary groups. As presented in Table 1, on average, participants were 32.11 years old, and more than half identified as Hispanic and never married. The most common substances used in the 8 months prior to treatment entry were methamphetamines, marijuana, and other drugs (alcohol to intoxication, cocaine or crack, and opiates). Notably, most participants reported use of multiple substances. The total possible range of reported traumatic experiences was 0–30. The mean number of traumatic events experienced across the lifetime was 13.59 (SD = 5.11).

**Table 1:** Sample characteristics (*N* = 241).

Variable	Frequency (%)
Race/Ethnicity	
Hispanic	141 (58.5)
Non-Hispanic Black	50 (20.7)
Non-Hispanic White	50 (20.7)
Marital status	
Married or common law	17 (7.1)
Separated, divorced, or widowed	45 (18.7)
Never married	179 (74.3)
Education	
Less than high school	115 (47.7)
High school or equivalent	67 (27.8)
Some postsecondary education	59 (24.5)
Employment in 8 months prior to treatment entry	
Full-time	33 (13.7)
Part-time	30 (12.4)
Not working	178 (73.9)
SUD diagnosis	
Alcohol use disorder (AUD)	23 (9.8)
Drug use disorder (DUD)	180 (76.6)
AUD and DUD	32 (13.6)
Substances used in 8 months prior to treatment entry	
(Meth)amphetamines	187 (77.6)
Marijuana	136 (56.4)
Alcohol to intoxication	120 (49.8)
Cocaine/Crack	33 (13.7)
Opiates (except heroin)	21 (8.7)
Other drugs	49 (20.3)
Top six specific life traumatic events	
Incarceration	202 (83.8)
Being homeless	186 (77.2)
Serious money problems (not enough money for food, rent)	185 (76.8)
Incarceration of close family member	170 (70.5)
Emotional abuse or neglect	165 (68.5)
	Mean (SD)
Age	32.11 (8.8)

Preliminary data screening indicated that all key variables in the models were normally distributed. Zero-order bivariate correlations among all key observed variables are presented in Supplementary Table S1. The examination of potential covariates indicated that age, education, housing, employment, and marital status were not significantly associated with emotion dysregulation or craving, whereas significant differences in both emotion dysregulation and craving were found by race and ethnicity. One-way analysis of variance results indicated significant differences in specific dimensions of emotion dysregulation, specifically lack awareness [*F*(2,238) = 5.42, *p* = 0.01] and emotional clarity [*F*(2,238) = 4.93, *p* = 0.01] by race and ethnicity. *Post hoc* comparisons revealed that Hispanic women on average reported higher scores on the emotion dysregulation dimension of lacking emotional awareness (*M* = 17.36, SD = 5.93) compared to their non-Hispanic Black counterparts (*M* = 14.36, SD = 5.58) and higher scores on lack of emotional clarity dimension (*M* = 13.05, SD = 4.59), compared to non-Hispanic White (*M* = 11.20, SD = 4.81) and non-Hispanic Black women (*M* = 11.18, SD = 4.02). Differences in alcohol and drug craving by race and ethnicity were also found [*F*(2,238) = 6.23, *p* = 0.02], with Hispanic women reporting higher levels of craving (*M* = 2.63, SD = 1.70) compared to non-Hispanic Black women (*M* = 1.66, SD = 1.41). Racial and ethnic differences in exposure to traumatic events were also evident [*F*(2,238) = 8.33, *p* < 0.001], with White women (*M* = 15.94, SD = 5.32) reporting greater exposure to number of traumatic events compared to Hispanic women (*M* = 12.64, SD = 4.50), with no significant differences among non-Hispanic Black women (*M* = 13.94, SD = 5.78). Lastly, levels of mindfulness disposition [*F*(2,238) = 5.26, *p* = 0.02] differed across race-ethnicity with Hispanic women reporting significantly lower levels of mindfulness disposition (*M* = 74.42, SD = 12.60) compared to non-Hispanic black women (*M* = 80.20, SD = 11.37, *p* = 0.02), and marginally significantly lower than non-Hispanic white women (*M* = 79.50, SD = 14.79, *p* = 0.051). Given the results of these preliminary findings, race and ethnicity was included as a covariate in all model testing during the primary analysis.

### Primary analyses

The first aim of this investigation was to examine the direct and indirect associations between CETE, emotion dysregulation and craving. As seen in Figure 1, the data revealed that greater CETE was associated with higher levels craving (*β* = 0.22, 95% CI: 0.09, 0.35, *p* = 0.001). A positive association between all dimensions of emotion dysregulation and craving was evident. It should also be noted that, a marginally significant indirect association was found whereby more CETE was associated with higher levels of craving *via* greater difficulties in engaging in goal-directed behaviors (*β* = 0.05, 95% CI = 0.001, 0.103). Results revealed that 2% of the variance in difficulties engaging in goal-directed behaviors was explained by CETE, whereas all predictor variables in the full model accounted for 19.6% of the variance in craving, *∆R*^2^ = 0.196, *F*(3, 241) = 19.23, *p* < 0.001.

The second study aim was to examine the direct and indirect associations between CETE, mindfulness disposition, and craving. As shown in Figure 2, greater CETE was associated with lower levels in the two mindfulness disposition dimensions of nonjudgment and awareness. Greater mindfulness disposition across all dimensions (except nonjudgment) was associated with more craving. Additionally, findings revealed an indirect associations between CETE and craving *via* the mindfulness dimension of acting with awareness, whereby greater CETE was associated with higher craving *via* lower levels of awareness (β = 0.03, 95% CI = 0.004, 0.074). Results of the mediation analyses indicated that 3.1% of the variance in awareness was explained by CETE, whereas all predictor variables in the full model accounted for 9% of the variance in craving, (*∆R*^2^ = 0.089, *F*(3, 241) = 7.70, *p* < 0.001).

The third study aim was to examine the moderating effect of mindfulness disposition on the associations between CETE, emotion dysregulation, and craving. As seen in Figure 3, a significant interaction effect between CETE and mindfulness disposition on craving was found (*β* = −0.14, *p* = 0.03; 95% CI = −0.27, −0.02). As seen in Table 2, conditional effects indicated that CETE was significantly associated with craving among women reporting low levels (1 SD below the mean; *β* = 0.32, *p* < 0.01; 95% CI = 0.14, 0.49), and average levels of mindfulness disposition *β* = 0.17, *p* = 0.01; 95% CI = 0.05, 0.30). However, no significant associations were evident between CETE and craving among women with higher levels (1 SD above the mean) of mindfulness disposition. No significant interaction effects were found between CETE and any dimension of emotion dysregulation or emotion dysregulation and craving. The plotted moderation effect is depicted in Figure 4.

**Table 2:** Conditional effects of cumulative exposure to traumatic events on craving across levels of mindfulness.

Level of mindfulness	Mean	Effect	Value of *p*	LLCI	ULCI
Low mindfulness	63.72	0.11	*p* < 0.001^***^	0.04	0.17
Average mindfulness	77.00	0.06	0.008^**^	0.02	0.10
High mindfulness	88.28	0.01	0.583	−0.04	0.07

^**^p < 0.01; ^***^p < 0.001; LLCI, 95% lower-level confidence interval; ULCI, 95% upper-level confidence interval.

## Discussion

To our knowledge, this is the first study to examine the associations between cumulative exposure to traumatic events, emotion dysregulation, and mindfulness disposition on alcohol and drug craving in a racially and ethnically diverse sample of women in SUD treatment. The moderating effect of mindfulness disposition on the associations among exposure to traumatic events, emotion dysregulation, and craving were also explored. Results from the current study partially support our hypotheses.

### Cumulative exposure to trauma events, emotion regulation, and craving

As expected, greater cumulative exposure to traumatic events was associated with higher levels of craving. Present study findings build on the limited existing studies conducted with vulnerable female populations (i.e., incarcerated women and those with SUD) that have found positive associations among trauma exposure, emotion dysregulation, and adverse mental health and substance use outcomes (Messina et al., 2014; Garland et al., 2019). However, these studies have focused primarily on interpersonal trauma such as physical and sexual abuse during childhood. Our results suggest the importance of addressing the cumulative effects of multiple and intersecting traumatic events (severe economic insecurity, exposure to violent crimes, involvement with the criminal justice system or child protective services, and homelessness) across the lifespan among women with SUD, as well as the role it may play in craving during the early phases of residential treatment.

Notably, rates of exposure to traumatic events in this sample were particularly high, which is consistent with other studies involving vulnerable women populations (Lynch et al., 2017). Indeed, every participant in the study sample reported experiencing at least one traumatic life event, with a mean of 14 traumatic events endorsed out of 30 possible events—considerably high given the relatively young age of the sample. Unexpectedly, White women reported greater exposure to traumatic events compared to Hispanic women. While it is possible that Hispanic women did indeed have lower levels of exposure to traumatic events, it could also be that Hispanic women were less likely to report traumatic events due to cultural factors associated with stigma, fears of being perceived as weak, or cultural expectations about what is or is not appropriate to share. Differences in trauma exposure between non-Hispanic White and Hispanic women may also be due to culturally specific events and structural barriers not captured in the present study. For instance, Hispanic women in first- and second-generation immigrant families are more likely to be exposed to immigration-related stressors such as fears of family member deportation and limited access to culturally responsive physical and mental health care services (Guerrero et al., 2013; Eghaneyan et al., 2020). These events were not captured in the Life Stressors Checklist used to assess exposure to traumatic events. Additionally, although the present study examined the cumulative impact of exposure to traumatic events, future research is needed to examine whether the types, severity, appraisal, and duration of specific traumatic experiences and the developmental point at which these events occurred (i.e., childhood versus adulthood or both) may have differential effects on emotion dysregulation and craving among women with SUD.

Study findings also revealed greater emotion dysregulation across all dimensions was positively associated with increased craving. These findings are in line with previous research linking emotional dysregulation with craving among individuals with SUD (e.g., Szasz et al., 2012; Khosravani et al., 2017) and corroborates this association in a sample of women in residential SUD treatment from diverse racial and ethnic backgrounds.

Results revealed a marginal positive association between exposure to traumatic events and the emotion dysregulation dimension of engaging in goal-directed behaviors. Indirect associations whereby greater exposure to traumatic events was linked with higher levels of craving *via* greater difficulties in engaging in goal-directed behaviors were also evident. Future studies with larger samples are needed to further explore the indirect associations found in the present study, as this may have clinical implications for the development of intervention strategies that promote goal directed behaviors during residential treatment among women with extensive histories of trauma exposure.

No other emotion dysregulation dimension was directly associated with women’s cumulative exposure to traumatic events. We posit that limited findings in significant associations between traumatic event exposure, emotion dysregulation and craving may be related to larger macro-level influences not captured by the Life Stressors Checklist. Findings from qualitative studies with Hispanic women in residential treatment (largely methamphetamine users such as those in the present study sample) suggest that perhaps larger social forces, including structural racism that permeates across multiple levels of influence, may play a stronger role in drug use (and overall drug use severity and craving), beyond the individual-level factors assessed in the present study (Cheney et al., 2018). Certainly, further examination is warranted regarding the intersection of structural and individual factors among women of color and its association with emotion dysregulation, mindfulness disposition, and substance use outcomes (such as craving) in this population.

Previous studies have examined associations among trauma, emotion dysregulation, and craving in relation to substances such as alcohol, tobacco, and heroin. Our study lays the foundation to inform how these associations may function in a sample consisting largely of methamphetamine users. Among used substances, methamphetamine is known to have the highest relapse and craving rates. It severely affects the brain’s dopamine system, creating an exceedingly high desire for use. This inevitably results in a cycle of use and cessation, with increased rates of relapse (Breese et al., 2011).

Although scientific understanding regarding targets of drug craving among women methamphetamine users in treatment is limited (e.g., Abdoli et al., 2019), the need to identify these factors in this population is pressing. A recent national historical trend analysis of patients admitted to SUD treatment between 1992 and 2017 found considerable increases in methamphetamine users in treatment, with a maximum growth rate of approximately 1,100% (higher than any other drug, including heroin, alcohol, marijuana, and cocaine or crack; (Mazumder et al., 2020). Unlike other drugs such as heroin or alcohol, which were predominantly endorsed by men, women in treatment were more likely to be methamphetamine users compared to men. Ethnic differences across methamphetamine users were also vast compared with other drugs. Methamphetamine users were 24% more likely to self-identify their ethnic origin as Mexican and 8 times more likely to identify as Cuban than Puerto Rican (Mazumder et al., 2020). Blacks and African Americans were 77% less likely to report methamphetamine use compared to Whites (Mazumder et al., 2020). To that end, the present study sample represents a growing priority group in SUD treatment.

### Cumulative exposure to traumatic events, mindfulness disposition, and craving

Our findings reveal that greater exposure to traumatic events were associated with lower mindfulness disposition dimensions of nonjudgment about inner experiences and acting with awareness. Several meta-analyses have examined associations between mindfulness disposition and psychosocial outcomes (Karyadi et al., 2014; Hanley and Garland, 2017; Carpenter et al., 2019). Meta-analytic findings show that acting with awareness, nonreactivity, and nonjudgment are most robustly associated with substance abuse behaviors (Karyadi et al., 2014) and negative affect (Carpenter et al., 2019). Our findings suggest that nonjudgment and acting with awareness are also negatively associated with cumulative exposure to traumatic events. This may be because the acting with awareness and nonjudgment facets of mindfulness disposition require higher-order cognitive processes (Ives-Deliperi et al., 2011), which have been shown to be compromised by trauma exposure (Pechtel and Pizzagalli, 2011). Although further research is warranted, our preliminary findings suggest that the promotion of nonjudgment and acting with awareness may be particularly fruitful targets among women in residential SUD treatment with extensive histories of trauma exposure.

Our findings also revealed that higher levels of exposure to traumatic events were indirectly associated with increased craving *via* lower levels of acting with awareness. Awareness of physical and emotional sensations and cues is thought to be critical for regulation, supporting healthy decisions and behavior change among individuals in SUD treatment (Price and Hooven, 2018). Current and evolving neurocognitive models highlight the importance of mindful awareness for the development of basic emotional awareness skills and the capacity for new emotion regulation appraisal and reappraisal processes that are critical for improved SUD treatment outcomes (Verdejo-Garcia et al., 2012). Recent randomized clinical trials examining the efficacy of integrating mindful awareness therapies as an adjunct to community-based SUD treatment have found increased emotion regulation, mindfulness skills, and improving substance use outcomes (including craving) among women with SUD. These studies have been conducted in largely White samples in the context of outpatient treatment (Verdejo-Garcia et al., 2012; Greenfield et al., 2018). Our findings suggest that these associations may also hold among racial and ethnically diverse women in residential treatment for SUD. As such, results from the present study build on previous research and suggest that promoting mindful awareness among racially and ethnically diverse women with extensive histories of trauma exposure may potentially curb craving during the early phases of residential treatment. Nevertheless, examining the effectiveness of mindful awareness-focused interventions in this population is necessary before further treatment implications can be posited.

### Moderating effects of mindfulness disposition

Our findings revealed a significant interaction effect between exposure to traumatic events and mindfulness disposition on craving whereby greater exposure to traumatic events was associated with higher levels of craving only among women with low and average (but not high) levels of mindfulness disposition. This suggests that dispositional mindfulness may have protective effects on craving among women with substantial histories of trauma exposure, while those with low levels of mindfulness disposition may be especially susceptible to high levels of craving early in residential treatment.

Previous evidence suggests that among racial and ethnic minoritized groups, mindfulness-based treatment approaches may be more effective than relapse prevention and lead to better drug use relapse outcomes compared to Whites, especially when an individual’s racial and ethnic minority status matches that of the majority of the clients participating in a mindfulness group intervention (Greenfield et al., 2018). As a present-centered treatment, mindfulness interventions may be also more aligned with the worldviews of racial-ethnic minoritized groups (Spears, 2019). Mindfulness-based interventions target-specific intra-and interpersonal stressors that may hinder progress of addiction treatment among disadvantaged populations (e.g., coping with discrimination, low self-efficacy, and low social support). These interventions also foster self-compassion and self-acceptance, which may be particularly valuable in the context of marginalization (Witkiewitz et al., 2013a; Iacono, 2019). The utility of mindfulness-based approaches with underserved and vulnerable populations is particularly noteworthy given that racial and ethnic minoritized groups are less likely overall to complete SUD treatment (Guerrero et al., 2013; Mennis and Stahler, 2016) and experience greater barriers to accessing treatment services, lower utilization rates, and less satisfaction with treatment compared to Whites (Guerrero et al., 2013).

Our results build on previous findings and suggest that integration of mindfulness-based interventions, particularly those that focus on awareness, may be beneficial for decreasing craving among women with SUD from diverse races and ethnicities with extensive histories of exposure to traumatic events. Nevertheless, our findings focus on mindfulness disposition, rather than treatment efficacy. Further research is necessary to examine the effectiveness of these approaches in curbing craving in this population. Specifically, future studies with larger sample sizes and sufficient power are needed to examine if racial and ethnic differences exist in these associations throughout stages of SUD treatment. Longitudinal research is also required to examine how associations between exposure to traumatic events, emotional dysregulation, mindfulness disposition, and craving may change throughout the treatment process.

It also important to note that while outside the specific aims of this investigation, our results revealed racial-ethnic differences across scores on various key study constructs. For instance, compared to their non-Hispanic White and non-Hispanic Black counterparts, Hispanic women reported higher average scores across two dimensions of emotion dysregulation, lack of emotional awareness and lack of emotional clarity. These results are consistent with findings from a recent systematic review that provide support for existing differences in emotion regulation across racial-ethnic-groups (Weiss et al., 2021). Researchers posit that worldviews, ideologies, values, and the concept of self vary across racial and ethnic groups and may influence how members of these groups evaluate, appraise, or react to emotional stimuli (Mennis and Stahler, 2016; Weiss et al., 2021). Specific emotion regulation strategies among minoritized racial-ethnic groups may reflect adherence to cultural norms related to emotional expression. Indeed, cultural values in collectivist (such as Asian and Hispanic cultures) and individualistic cultures have been tied to emotion regulation strategies, with individualist cultures preferring expression of emotions and collectivistic cultures being more likely to apply emotional suppression (Ramzan and Amjad, 2017). As such, future research that identifies how specific cultural factors (e.g., ethnic identity, acculturation, and values adherence) may influence racial and ethnic differences in emotion regulation is warranted. Additionally, investigations into treatments that incorporate emotion regulation strategies that align with racial and ethnic cultural worldviews, ideologies, and values may serve to bolster substance use treatment outcomes among racial-ethnic minoritized populations.

Lastly, our findings identifying evident differences by race-ethnicity across key study constructs (i.e., mindfulness, emotion dysregulation, and craving) may also be a function of the psychometric properties of the utilized scales. Recent studies have revealed significant differences in item functioning across scales assessing affect and trauma among underserved groups such as racial and ethnic minoritized populations (i.e., the Clinician-Administered PTSD Scale for *DSM–IV* (CAPS-IV; Ruglass et al., 2020; Morgan-López et al., 2022) Future studies are needed to examine if these similar psychometric issues are present in other widely used scales such as those utilized in the present study. Indeed, these differences may likely have implications for treatment interventions that target racial-ethnic minoritized groups with substance use dependence.

### Limitations

Findings from this investigation should be interpreted in light of its limitations. The cross-sectional data limit causal inferences and interpretation of mediating effects. However, it is important to note the chronological nature of the examined constructs in the research design. The independent variable of interest in the present study was collected retrospectively based on past exposure to traumatic events, whereas the mediator, moderator, and outcomes were collected based on the past 30 days, implying the foundation for temporal ordering and adding support for utilization of the current design (Shrout, 2011; Hayes et al., 2017). Nevertheless, we frame our findings in terms of indirect association and suggest that the present study findings provide theoretical contributions and lay the foundation for further longitudinal mediation analysis of the key study constructs. It is also possible that bidirectional associations exist between emotional regulation, mindfulness disposition and craving. For instance, stronger craving may hinder an individual’s appraisal or capacity to regulate emotions or implement mindfulness practices. While testing bidirectional associations of these constructs was outside the scope investigations it is important to note that future research exploring these relationships is warranted. Additionally, the self-report measures may be prone to social desirability bias, and retrospective reporting may contribute to false negative reports of early adverse childhood events (Hardt and Rutter, 2004). Self-reported alcohol and drug craving may be susceptible to biases of retrospective reporting (Serre et al., 2015). The nonreactivity subscale of the mindfulness scale also demonstrated lower internal consistency in this sample. Future research should develop a scale that more accurately captures the aspect of remaining nonreactive to internal experiences among diverse women with SUD. While bivariate differences across race-ethnicity were found across the key constructs in the present study, the limited sample size precluded testing whether race-ethnicity served as a modifier in the research model. An additional limitation were the relatively small effect sizes found in the present study. Previous meta-analyses have also noted small effect sizes on studies examining associations between mindfulness disposition and substance use behaviors (Karyadi et al., 2014). Despite the small mindfulness disposition–craving association, it is possible that mindfulness could still be an important target in treatment, particularly as it relates to the dimension of acting with awareness serving as an indirect pathway in the association between trauma exposure and craving, as found in the present study. Future studies with larger sample sizes are needed to further examine the clinical utility of integrating these strategies in SUD treatment among women with extensive histories of trauma exposure. Lastly, findings are based on a sample of women with SUD in residential treatment and may not be generalized to men or women being treated in outpatient SUD programs.

### Conclusion

The present study contributes to understanding the associations among cumulative exposure to traumatic events, emotion dysregulation, mindfulness disposition, and substance use craving among racially and ethnically diverse women with SUD. While mindfulness disposition is considered a trait, it can be enhanced through mindfulness training, such as that provided by mindfulness-based interventions (Kiken et al., 2015). The buffering effect that mindfulness disposition had on the association between traumatic event exposure and craving warrants further examination into the potential benefits of integrating these mindfulness strategies into SUD treatments for women with extensive histories of exposure to traumatic events. Future research replicating these results in larger samples is needed to further examine these constructs as beneficial treatment targets among diverse racial and ethnic women in SUD interventions.

## Data availability statement

The raw data supporting the conclusions of this article will be made available by the authors, without undue reservation.

## Ethics statement

The studies involving human participants were reviewed and approved by the University of Southern California Institutional Review Board (IRB; UP-14-00391). The patients/participants provided their written informed consent to participate in this study.

## Author contributions

MS and HA contributed to the conceptualization, writing of the manuscript, and provided suggested revisions and feedback throughout the writing process. MS conducted the data analysis, and HA was the principal investigator of the study. All authors contributed to the article and approved the submitted version.

## Funding

This research was supported by National Institute on Drug Abuse (Grant #5R01DA038648, PIs: Hortensia Amaro and David Black) cosponsored by the National Institute on Alcohol Abuse and Alcoholism. Support was also provided by Grant #5S21MD010683-05 from the National Institutes of Minority Health and Health Disparities (NIMHD). The ideas and opinions expressed herein are those of the authors and endorsement of those opinions by funders is not intended nor inferred.

## Conflict of interest

The authors declare that the research was conducted in the absence of any commercial or financial relationships that could be construed as a potential conflict of interest.

## Publisher’s note

All claims expressed in this article are solely those of the authors and do not necessarily represent those of their affiliated organizations, or those of the publisher, the editors and the reviewers. Any product that may be evaluated in this article, or claim that may be made by its manufacturer, is not guaranteed or endorsed by the publisher.
