# Supplementary material for: Cumulative exposure to traumatic events and craving among women in residential treatment for substance use disorder: The role of emotion dysregulation and mindfulness disposition
Source: Front Psychol. 2022 Nov 24;13:1048798. doi: 10.3389/fpsyg.2022.1048798 (PMC9730726; doi:10.3389/fpsyg.2022.1048798)
Supplement: Supplementary file 1 [file Table_1.DOCX]

|  | |  |  |  |  |  |  |  |  |  |  |  |  |  |  |  |  |  |  |  |  |  |
| --- | --- | --- | --- | --- | --- | --- | --- | --- | --- | --- | --- | --- | --- | --- | --- | --- | --- | --- | --- | --- | --- | --- |
| Supplementary Table 1. Bivariate correlations of key study constructs. | | | | | | | | | | | | | | | | | | | | | |  |
|  | Variable | | | M | SD | Range | 1 | 2 | 3 | 4 | 5 | 6 | 7 | 8 | 9 | 10 | 11 | 12 | 13 | |  |  |
| 1. | Craving | | | 2.41 | 1.72 | 1-6 | ­– |  |  |  |  |  |  |  |  |  |  |  |  | | | |
| 2. | CETE | | | 13.59 | 5.11 | 0-36 | .19^**^ | – |  |  |  |  |  |  |  |  |  |  |  |  | | |
| Emotion regulation | | | |  |  |  |  |  |  |  |  |  |  |  |  |  |  |  |  | | | |
| 3. | Strategies | | | 18.41 | 7.96 | 8-40 | .41^**^ | .08 | – |  |  |  |  |  |  |  |  |  |  | | | |
| 4. | Non-accept. | | | 14.26 | 6.57 | 6-30 | .35^**^ | .09 | .77^**^ | – |  |  |  |  |  |  |  |  |  | | | |
| 5. | Goals | | | 13.40 | 5.36 | 5-25 | .40^**^ | .12 | .78^**^ | .66^**^ | – |  |  |  |  |  |  |  |  | | | |
| 6. | Impulse | | | 14.31 | 6.27 | 6-30 | .36^**^ | .03 | .77^**^ | .60^**^ | .78^**^ | – |  |  |  |  |  |  |  | | | |
| 7. | Awareness | | | 16.38 | 5.94 | 6-30 | .27^**^ | -.01 | .44^**^ | .33^**^ | .36 | .38^**^ | – |  |  |  |  |  |  | | | |
| 8. | Clarity | | | 12.28 | 4.60 | 5-25 | .31^**^ | .01 | .65^**^ | .60^**^ | .58^**^ | .59^**^ | .61^**^ | – |  |  |  |  |  | | | |
| Mindfulness | | |  |  |  |  |  |  |  |  |  |  |  |  |  |  |  |  |  |  |  |  |
| 9. | Describe | | | 16.54 | 4.27 | 5-25 | -.22^**^ | -.04 | -.42^**^ | -.34^**^ | -.39^**^ | -.38^**^ | -.52 | -.64^**^ | – |  |  |  |  | | | |
| 10. | Nonreact. | | | 14.90 | 3.97 | 5-25 | -.18^**^ | -.01 | -.25^**^ | -.13^*^ | -.24^**^ | -.30^**^ | -.27^**^ | -.31^**^ | .35^**^ | – |  |  |  | | | |
| 11. | Observe | | | 13.73 | 3.95 | 4-20 | -.15^*^ | -.06 | -.15^*^ | -.05 | -.15^*^ | -.16^*^ | -.42^**^ | -.36^**^ | .39^**^ | .52^**^ | – |  |  | | | |
| 12. | Non-judge. | | | 14.63 | 4.40 | 5-25 | -.10 | -.17^**^ | -.32^**^ | -.40^**^ | -.22^**^ | -.12 | -.14^*^ | -.28^**^ | .21^**^ | -.20^**^ | -.23^**^ | – |  | | | |
| 13. | Act aware | | | 16.87 | 4.76 | 5-25 | -.23^**^ | -.14^*^ | -.59^**^ | -.46^**^ | -.56^**^ | -.50^**^ | -.44^**^ | -.51^**^ | .47^**^ | .05 | .13^*^ | .46^**^ | – | | | |

Note. * p<.05; **p<.01
